# Analysis of the microRNA signature in left atrium from patients with valvular heart disease reveals their implications in atrial fibrillation

**DOI:** 10.1371/journal.pone.0196666

**Published:** 2018-05-03

**Authors:** Rosa Doñate Puertas, Audrey Jalabert, Emmanuelle Meugnier, Vanessa Euthine, Philippe Chevalier, Sophie Rome

**Affiliations:** 1 Institut NeuroMyoGene (INMG), UMR CNRS 5310-INSERM U1217 / University of Lyon, Lyon, France; 2 CarMeN Laboratory (UMR INSERM 1060-INRA 1397, INSA), Lyon-Sud Faculty of Medicine, University of Lyon, Pierre-Bénite, France; 3 Rhythmology Unit, Louis Pradel Cardiology Hospital, Hospices Civils de Lyon, Bron, France; Universitat de Barcelona, SPAIN

## Abstract

**Background:**

Among the potential factors which may contribute to the development and perpetuation of atrial fibrillation, dysregulation of miRNAs has been suggested. Thus in this study, we have quantified the basal expressions of 662 mature human miRNAs in left atrium (LA) from patients undergoing cardiac surgery for valve repair, suffering or not from atrial fibrillation (AF) by using TaqMan^®^ Low Density arrays (v2.0).

**Results:**

Among the 299 miRNAs expressed in all patients, 42 miRNAs had altered basal expressions in patients with AF. Binding-site predictions with Targetscan (conserved sites among species) indicated that the up- and down-regulated miRNAs controlled respectively 3,310 and 5,868 genes. To identify the most relevant cellular functions under the control of the altered miRNAs, we focused on the 100 most targeted genes of each list and identified 5 functional protein-protein networks among these genes. Up-regulated networks were involved in synchronisation of circadian rythmicity and in the control of the AKT/PKC signaling pathway (*i*.*e*., proliferation/adhesion). Down-regulated networks were the IGF-1 pathway and TGF-beta signaling pathway and a network involved in RNA-mediated gene silencing, suggesting for the first time that alteration of miRNAs in AF would also perturbate the whole miRNA machinery. Then we crossed the list of miRNA predicted genes, and the list of mRNAs altered in similar patients suffering from AF and we found that respectively 44.5% and 55% of the up- and down-regulated mRNA are predicted to be conserved targets of the altered miRNAs (at least one binding site in 3’-UTR). As they were involved in the same biological processes mentioned above, these data demonstrated that a great part of the transcriptional defects previously published in LA from AF patients are likely due to defects at the post-transcriptional level and involved the miRNAs.

**Conclusions:**

Our stringent analysis permitted us to identify highly targeted protein-protein networks under the control of miRNAs in LA and, among them, to highlight those specifically affected in AF patients with altered miRNA signature. Further studies are now required to determine whether alterations of miRNA levels in AF pathology are causal or represent an adaptation to prevent cardiac electrical and structural remodeling.

## Background

Atrial fibrillation (AF) is the most common cardiac arrhythmia encountered in clinical practice [1]. It is characterized by irregular impulses to the ventricles and is the major cause of embolic stroke [1, 2]. AF is associated with atrial remodeling of the left atrium (LA) [3, 4] and it is not clear whether AF is a final result of atrial remodeling induced by heart diseases combined with deleterious environmental factors [5] or is a major cause of remodeling which perpetuates its progression. Over the last decade, the understanding of the mechanisms involved in AF has advanced tremendously and a number of genetic [6–8] and epigenetic factors [9–11] have been identified. In that context, microRNAs (miRNAs) have been recently added in the list of molecular actors associated with AF development [11–17], particularly in the 2 fundamental components of atrial remodeling which are electrical remodeling (*i*.*e*. alteration of ion channels) [18–22] and structural remodeling caused by fibrosis [12, 23–28]. MiRNAs are new regulators, biomarkers and therapeutic targets of cardiac pathologies [29–31]. They represent a class of evolutionally conserved small non-coding RNAs which function as negative regulators of gene expression [32]. Mature miRNAs regulate gene expression post-transcriptionally by binding to target mRNAs in association with the multiprotein RNA induced silencing complex [33]. Three mechanisms have been described for gene regulation via miRNA: (i) translation repression [32], (ii) direct mRNA degradation [34], and (iii) miRNA-mediated mRNA decay [35]. As these small RNAs can regulate multiple target mRNAs and individual mRNA can be targeted by several miRNAs, it is not surprising that miRNA deregulation is a hallmark of several pathological conditions. In the context of AF, a group of miRNAs have been identified for being able to be involved in AF as regulators of ion-channel proteins [36] or to be related to AF development, based on their altered basal expression in atria of patient suffering from AF [37, 38]. Recently, we have performed a transcriptomic analysis on human LA from patients with valvular heart disease (VHD) associated with or without AF in order to identify the transcriptional signature specifically related to the AF condition and demonstrated that promoter hypermethylation was associated to some of these transcriptional alterations [39]. In order to determine whether miRNA dysregulation could also be associated with heart remodeling associated with AF development (*i*.*e*. left atrial dilatation and interstitial fibrosis) we have quantified the miRNA levels of 662 different mature human miRNAs in LA of AF patients by using the TaqMan^®^ Low Density array (v2.0). Among the 299 miRNAs expressed in all patients 42 miRNAs had altered basal expressions confirming the involvement of miRNAs in the pathology. In silico predictions suggested that they were involved in several important biological processes and functional pathways associated with AF, such as TGF-beta 1 and Insulin-like growth factor 1 (IGF-1) signaling, proliferation and cellular adhesion (MAPkinase and AKT pathways), and circadian clock/synchronisation of circadian rhythmicity, but also in RNA-mediated gene silencing (*i*.*e*. AGO/EIF2C proteins) and miRNA machinery (*i*.*e*. TNRC6 proteins), further suggesting that alteration of miRNAs in LA would in fact perturbate the whole miRNome. Further bioinformatic analyses suggested that these altered miRNAs might explain part of the altered transcriptional signature previously identified as associated with the pathology [39], as altered miRNA were predicted to target half of the altered mRNA in LA of AF patients.

## Methods

### Patient characteristics

Many cardiovascular diseases predispose to AF. However, heart tissues from AF patients are usually compared to tissues from healthy subjects, which could mask the identification of alterations specifically related to AF (a left atrial disease), compared with other associated cardiac diseases. Therefore, to identify a miRNA signature relevant for AF pathology and not for valvular heart disease, using controls suffering from a unique cardiac pathology was critical. Samples from LA or pulmonary veins (PVs)-LA junctions were obtained from 8 patients undergoing cardiac surgery for valve repair at the Louis Pradel Cardiology Hospital (Hospices Civils de Lyon, Bron, France). Tissue samples were snap-frozen in liquid nitrogen and immediately stored at -80°C. This study was approved by the ethics committee of Lyon and informed consent was obtained from each patient (DC2015-2566). Patient clinical characteristics are provided in Table 1. Among the 8 participants, 4 were suffering from AF. No significant differences for medication or body mass index (BMI) were observed between patients with and without AF.

**Table 1:** Patients metabolic parameters, enrolled for miRNA quantification in left atria.

		Patients with AF	Control group
	**Number of patients**	4	4
	**Ratio M/F**	0/4	2/2
	**Age (year)**	74.3 (± 5.3)	56 (± 13)
	**BMI**	27.9 (± 4.9)	29.7 (± 5.1)
**Associated pathologies**	**Diabetes**	2	0
	**Dyslipidemia**	1	1
	**Hypertension**	2	4
**Age of AF onset**		62.5 (± 5.8)	NA
**AF Type**	**Parosxysmal**	0	NA
	**Persistent**	2	NA
	**Permanent**	2	NA
**LA surface (cm2)**		41 (± 8.1)	23 (± 5.7)*
**LVEF (%)**		54 (± 8.1)	70 (± 6.5)*
**Medications**	**Statine**	1	1
	**ACE/ARA II**	0	2
	**Betablocker**	2	0
	**Diuretics**	4	2
	**Calcium blocker**	2	1

* = p<0.05 (Patients with AF vs Control group; Student’ t test)

### MiRNA profiling

Total RNA were extracted from heart biopsies with mirVana^™^ miRNA Isolation Kit (Ambion, AppliedBiosystems). We quantified the expression of 662 different mature human miRNAs (Sanger miRBase v14) by using TaqMan^®^ Low Density array v2.0 with the Applied Biosystems 7900HT Fast Real time polymerase chain reaction (PCR) system equipped with a 384 well reaction plate [40]. 150ng of total RNA was used for reverse transcription using Megaplex^™^ RT Primers. A preamplification reaction with Megaplex^™^ PreAmp Primers was performed before the PCR reactions. Real time PCR was performed using standard conditions. Only miRNA expressed in the 8 patients were selected for further analysis (n = 315). To reduce bias caused by using an arbitrary miRNA as normalization correction factor, the miRNA expressions were compared between FA patients and controls based on their relative expression to the overall miRNA expression on each array using mean normalization analysis. *p*-values (Unpaired Student’s *t*-test) were calculated to test the difference of specific miRNA based on threshold cycle (Ct) values.

### Hierarchical clustering

Hierarchical clustering was calculated by using pairwise average-linkage clustering and drawn by using GenePattern (v3.2.3, http://pwbc.garvan.unsw.edu.au/gp, [41, 42]).

### Bioinformatic analysis

MiRNA target gene predictions are from Targetscan 7.0 (http://www.targetscan.org/vert_70/) [43] which predicts biological targets of miRNAs by searching for the presence of 8mer, 7mer, and 6mer sites that match the seed region of each miRNA [44]. Prediction of miRNA functions were determined by using DIANA-miRPath v3. 0 (http://www.microrna.gr/miRPathv3) [45]. Functional protein association networks were identified by using STRING 10.5 (https://string-db.org/) [46]. Cellular KEGG pathway enrichments were determined by using BABELOMICS 4.3 (http://v4.babelomics.org/) [47].

### Cell culture

To validate the binding of candidate miRNA on predicted target genes, the human embryonic kidney 293T cell line (HEK293T, ATCC^®^ CRL-1573^™^) was used. Cells were grown in Dulbecco’s modified Eagle’s medium (DMEM, PPA laboratories) containing 4,5 g/l glucose, 10% fetal bovine serum, 1% penicillin and streptomycin (PPA laboratories) and 1% of glutamine, at 37 °C in a humidified atmosphere of 5% CO2. Exponentially growing cells at 80% confluence were used for all the experiments.

### Dual-luciferase reporter assay

The 3’-UTR region of *Smyd2* was cloned in pEZX-MT01, a firefly/Renilla Duo-Luciferase reporter vector from GeneCopoeia (#HmiT054794, Inc., Rockville, MD, USA). MiR-519a-3p or miR-519b-3p precursors were cloned in pEZX-MR04 vector also expressing GFP (#Hmi R0453; #Hmi R0320, GeneCopoeia^™^, Inc., Rockville, MD, USA). HEK293T cells were plated at a density of ~3x10^5^ cells per well in 12-well plates. Two days later, cells were transfected in quadruplicate with *Smyd2* 3'-UTR sequence concomitantly with either miR-519a-3p or miR-519b-3p precursors or an empty pEZX vector used as control, by using ExGen500 transfection reagent (EUROMEDEX). After 48h, both Firefly luciferase and Renilla luciferase activities were measured on cell lysate using the Dual-Glo^®^ Luciferase Reporter Assay System (PROMEGA) with a GLOMAX^™^20/20 luminometer (Promega) with an acquisition time set up at 10 seconds. Values for Firefly luciferase activities were normalized against Renilla luciferase activity values for each transfected well.

### MiR-519b-3p overexpression

Transfections were carried out using ExGen500 transfection reagent (Euromedex) following the manufacturer´s protocol. 80% confluent HEK293T cells were transfected with pre-miR-519b-3p using a 1:5 ratio (μg DNA/μl ExGen500 reagent). After 48h, total RNA from transfected cells were extracted by using TRIzol^®^ Reagent (T9424, Sigma-Aldrich) and *Smyd2* mRNA level was quantified by qRT-PCR with 1 μg of total RNA (Sens_5´AGGCAGAAGCCATCCGAGAC; Antisens_5´ATGGCCTGGTACATCATGTG) on a Rotor-Gene 6000system (Corbett Life Science, Paris, France) using SYBER Green (Thermo Scientific). Relative changes in *Smyd2* were estimated from the Ct value and normalized to the respective Ct value of TATA box binding protein (TBP) determined in corresponding samples.

### Statistical analysis

Data are expressed as means ± SEM. For pairwise comparisons, Student’s *t*-test was used. Differences were considered statistically significant when *p*<0.05.

## Results

### miRNAs are involved in tissue maintenance and structure in human LA

**Fig 1:**
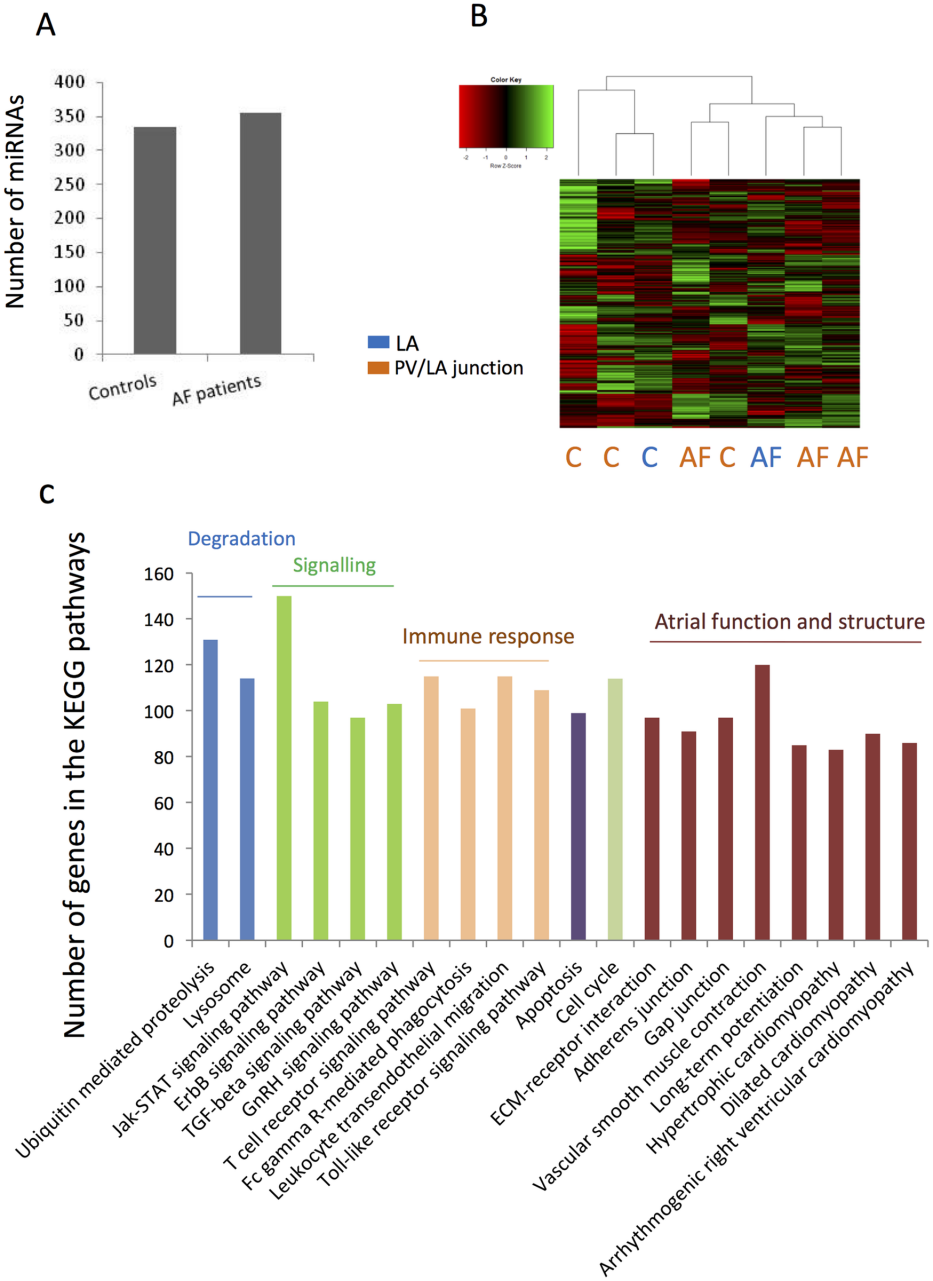
miRNA expression in human left atria (LA) from patients suffering from valvular heart disease (VHD patients) associated with atrial fibrillation (AF patients) or without AF (controls). A) Number of miRNA expressed in LA from controls or AF patients. B) Unsupervised hierarchical clustering and heat-map of the 299 miRNA basal expressions, expressed in all VHD patients. LA = biopsies from LA; PV/LA = biopsies from pulmonary vein-LA junction. C) Significantly enriched KEEG pathways (BABELOMICS analyses) with target genes of the 299 miRNAs (prediction from TARGETSCAN). Only pathways containing more than 50 predicted genes were considered.

**Fig 2:**
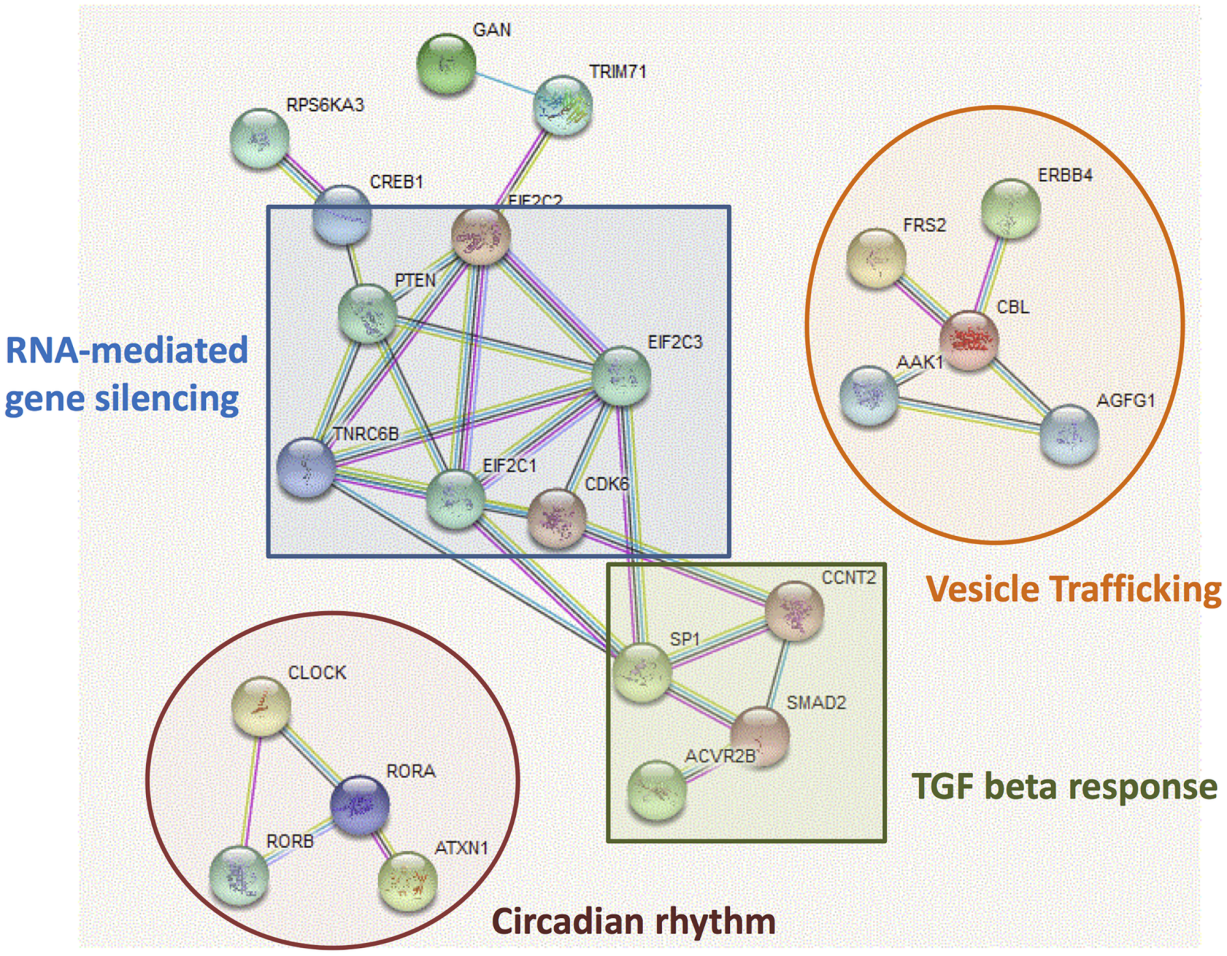
Protein-protein interactions between the 100 most targeted genes by the 299 miRNAs expressed in left atria from patients suffering from valvular heart disease. Predicted protein-protein interactions are from STRING. The interactions include direct (physical) and indirect (functional) associations either from computational predictions or from knowledge transfer between organisms or from interactions aggregated from other (primary) databases. The list of genes is given in S2 Table.

Tissues from LA or from PV- LA junction were obtained from 8 patients suffering from VHD with (n = 4) or without (n = 4) AF. There were no significant differences in terms of age or BMI between the 2 groups. Doppler echocardiographies showed that LA size of patients with AF was significantly greater than patients without AF as previously reported [39]. The levels of 662 mature miRNAs were quantified. 356 were expressed in AF patients and 335 in control subjects (Fig 1A) and 299 miRNAs were expressed in all patients (Fig 1B and S1 Table). Thus conversely to the study of Liu et al. 2014 [48], we did not confirm that AF affects significantly the number of expressed miRNAs in LA. The 299 common miRNAs were predicted to bind 129,703 binding sites in 3’-UTR of 11,884 target genes (prediction from Targetscan, conserved family) (S2 Table). KEGG pathways with significant miRNA target-gene enrichment were involved in protein degradation, signaling pathways, apoptosis, and atrial function and structure (*e*.*g*.; gap junction and contraction) (Fig 1C). Interestingly, the 299 common miRNAs were predicted to interact with 750 mRNA coding for transcription factors (G.O molecular function: transcription factor activity (GO: 0003700), *p* = 2.76518E-42, prediction from BABELOMICS, data not showed) supporting the evidence of miRNAs as key components of the transcriptional signature in LA. As it is known that individual miRNA may reduce modestly target gene expression while a collection of miRNAs may act in a combinatorial way to exert significant effect, we further focused on the 100 most targeted genes (S2 Table) in order to identify the most relevant cellular functions of the 299 miRNAs in human LA. Based on published and predicted protein-protein interactions [46], we identified 4 important functional protein-protein networks, involving 22 highly targeted genes related to RNA-mediated gene silencing and miRNA machineries, vesicle trafficking, response to Transforming growth factor (TGF)-beta and regulation of circadian rhythm (Fig 2). Thus many of these functions, which are known to be tightly regulated in order to maintain cardiac function to prevent fibrosis and hypertrophy [49] or to control blood pressure and heart rate [50], are predicted to be under the controlled of miRNAs in LA. Interestingly, we found that proteins involved in RNA-mediated gene silencing (*i*.*e*. AGO/EIF2C proteins) and miRNA machinery (*i*.*e*. TNRC6 proteins) are also highly targeted by miRNAs suggesting that alteration of miRNAs in LA would perturbate miRNA functioning, in general.

### AF is associated with miRNAs with impaired basal expressions in human LA

**Fig 3:**
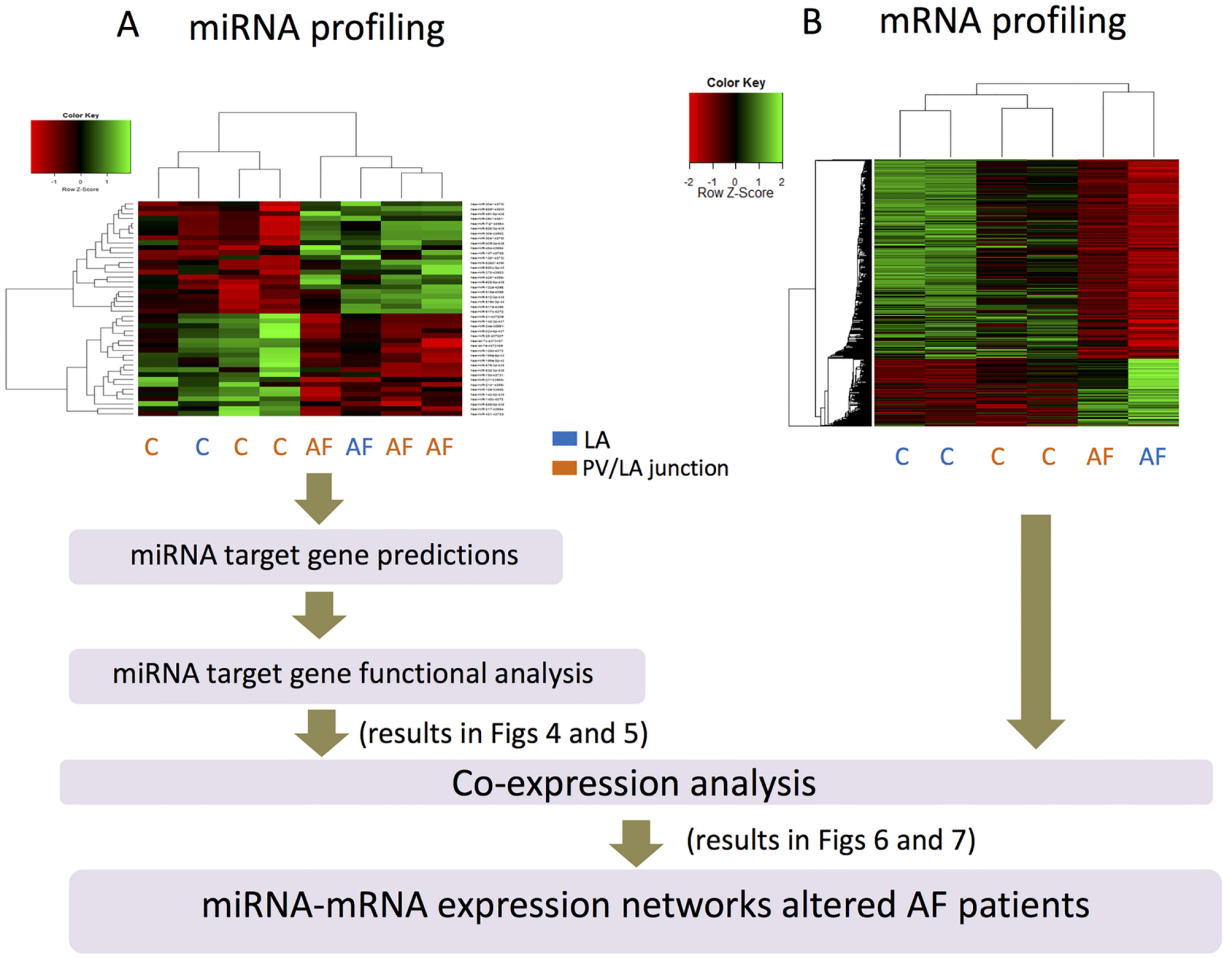
Workflow use to predict the function of the 42 altered miRNAs in left atria (LA) from patient suffering from atrial fibrillation (AF). A) Unsupervised hierarchical clustering and heat-map of the 42 miRNA basal expressions, expressed in patients suffering from valvular heart disease associated with AF or without AF (C = Controls). LA = biopsies from LA; PV/LA = biopsies from pulmonary vein-LA junction. B) mRNA profiling data are from [39] and are available from the Gene Expression Omnibus database GSE31821 (www.ncbi.nlm.nih.gov/gds/).

**Fig 4:**
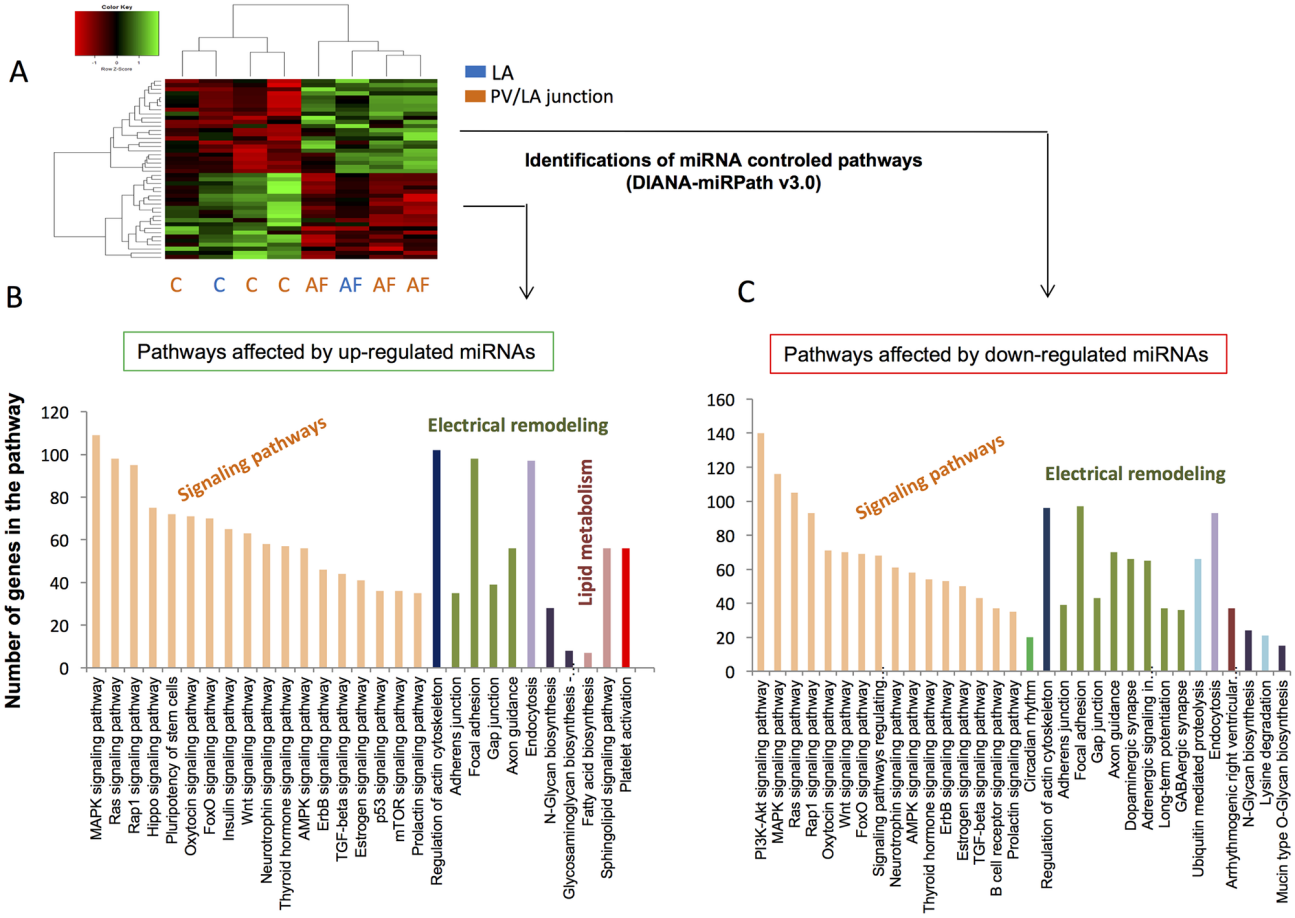
Predictions of miRNA controlled pathways affected by the up- or down-regulated miRNAs in left atria (LA) from patients suffering from atrial fibrillation (AF). A) Unsupervised hierarchical clustering and heat-map of the 42 miRNA basal expressions, altered in patients with AF or without AF (C = Controls). LA = biopsies from LA; PV/LA = biopsies from pulmonary vein-LA junction. B) and C) Significantly enriched KEEG pathways with target genes of the 42 altered miRNAs. Pathway predictions are from DIANA-miRPath v3.0 [45].

As miRNAs expressed in LA were predicted to regulate important pathways for LA tissue maintenance and structure, we postulated that altered miRNA basal expressions might be associated with AF. We identified 42 dysregulated miRNAs in patients suffering from AF (n = 4) (S4 Table) compared to non AF patients, used as controls (n = 4). Because this result was not correlated with the origin of the tissue (LA *vs* /PV—LA junction) we consider that these miRNAs are related to the AF condition (Figs 3A and 4A). In order to understand the origin of their dysregulations, we first analyzed their genomic locations. Nine of the altered miRNAs were located on chromosome 19 which has the highest gene density of all human chromosomes. Interestingly chromosome 19 contains genes directly implicated in cardiomyopathy, muscular dystrophy congenital 1C, muscular dystrophy limb-girdle type 2I, muscular dystrophy with rimmed vacuoles and myotonic dystrophy [51]. Therefore, our data suggests that the non-coding part of this chromosome is also associated with muscular alterations; *i*.*e*. atrial fibrillation. Twenty % of the up-regulated miRNAs were located on chromosome 17, which has not been related to muscular or heart pathologies until now. Then, as it was suggested in the literature that intronic miRNAs are regulated similarly to their host genes [52–54] we determined whether it was the case for the dysregulated intronic miRNAs, by using our previous transcriptomic analysis on LA from patients suffering from AF [39]. Four miRNAs had similar altered expressions as their host genes (miR-126-5p, miR-589-3p, miR-15b-5p, miR-25-3p). This data indicated that in fact the majority of the altered intronic miRNAs had their proper promoters under the control of specific transcription factors, which might be altered in AF. In line with this hypothesis, promoter hypermethylation of *Pitx2* [39] is associated with its down-regulation in LA from patients with AF [55], and could explain the down-regulation of miR-133a (S3 Table), which is under PITX2 control [56]. Moreover during the past decade, a number of mutations in transcription factors have been reported to underlie congenital heart diseases associated with structural cardiac defects [57–59]. Finally, as about half of miRNA coding genes are associated with CpG islands [60], it could be envisaged that the 42 dysregulated miRNA represent candidate targets of the DNA methylation machinery. For instance, hyper- and hypomethylations have been associated with the down- and up-regulations of miR-378 and miR-25, respectively, in hepatocellular carcinoma cells [61]. Interestingly, these 2 miRNAs are also up- and down-regulated, respectively, in LA from AF patients (S3 Table). Furthermore miR-126, let-7a, miR-34a and miR-148, which are in the list of the 42 altered miRNAs, are frequently hypermethylated in cancers [62–65], an epigenetic regulatory feature which significantly participates in AF [9, 39].

### Altered miRNA profile is correlated with genes that are involved in AF aetiology

In order to determine the functional consequences of the 42 miRNA altered expressions in human LA suffering from AF, we followed the workflow presented on Fig 3. Target gene predictions indicated that the up- and down-regulated miRNAs targeted respectively 3,310 and 5,868 unique genes, with an overlap of 1,562 common genes (S4 Table). The significantly gene-enriched KEGG pathways are indicated on Fig 4A and 4B. These pathways were mainly signaling pathways involved in proliferation, morphogenesis and in the regulation of pluripotency of stem cells and pathways involved in electrical remodeling, endocytosis, glycan biosynthesis and in the regulation of actin cytoskeleton. Up-regulated miRNAs were predicted to specifically down-regulate pathways of the lipid metabolism and platelet activation; down-regulated miRNAs were specifically predicted to up-regulate pathways for protein degradation and arrhythmia.

**Fig 5:**
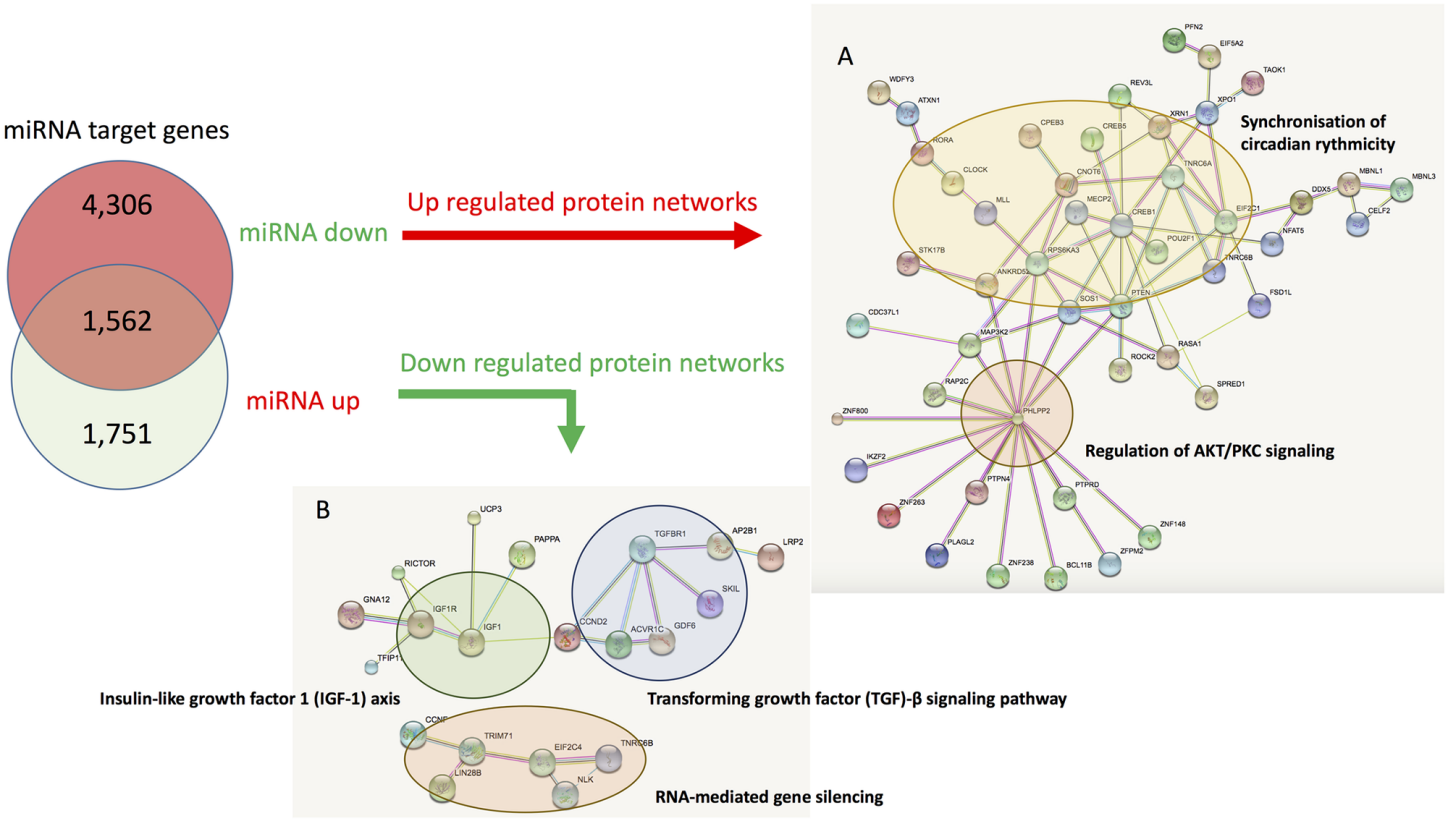
Protein-protein interactions between the 100 predicted most targeted genes, either by the up- or down-regulated miRNAs in left atria from patients suffering from valvular heart disease with or without atrial fibrillation. MiRNA target gene predictions are from TARGETSCAN. Predicted protein-protein interactions are from STRING.

Among the 100 most targeted genes of the 2 lists, in terms of miRNA binding sites (S4 Table), 5 functional protein-protein networks were predicted to be affected by the dysregulated miRNAs (Fig 5). Predicted up-regulated protein networks were involved in synchronisation of the circadian rythmicity and in the control of the AKT/PKC signaling pathway which is known to regulate the balance between cell survival and apoptosis (Fig 5A). Predicted down-regulated pathways were the IGF-1 pathway (involved in the regulation of contractility, glucose metabolism, hypertrophy, autophagy, senescence, and apoptosis), the TGF-beta signaling pathway (involved in cardiac hypertrophy and fibrosis) and a network involved in RNA-mediated gene silencing (Fig 5B).

**Fig 6:**
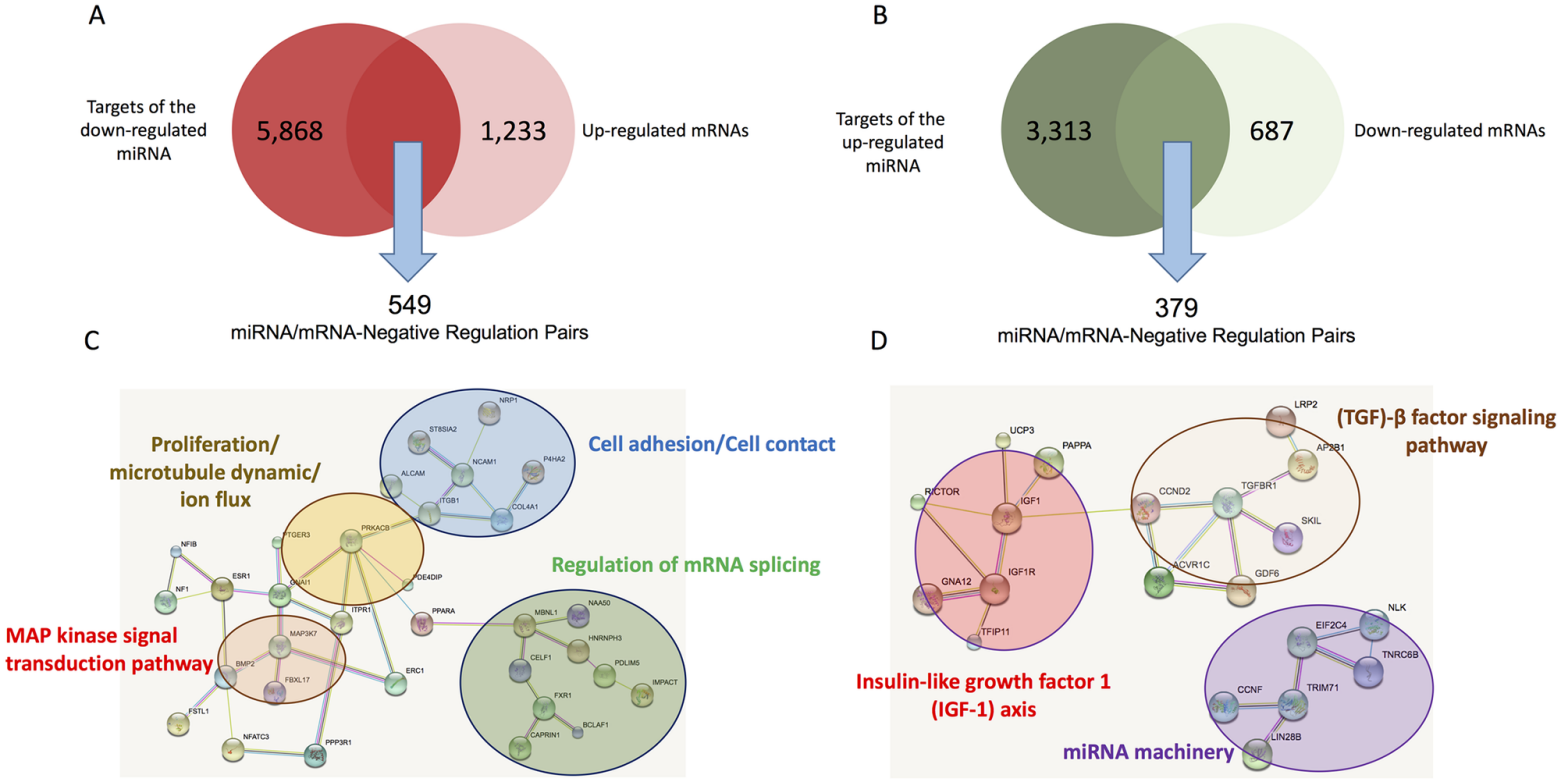
Inverse correlations between altered miRNAs and mRNA expressions in left atria from patients suffering from valvular heart disease with or without atrial fibrillation. Predictions are from TARGETSCAN. A) Data crossing between target gene predictions of the down-regulated miRNAs and the up-regulated mRNA from transcriptomic analyses. B) Data crossing between target gene predictions of the up-regulated miRNAs and the down-regulated mRNA from transcriptomic analyses. (C) and (D), the 100 most miRNA-targeted genes from the 2 lists (549 and 379) were analysed by using STRING to determine whether they were in included in protein-protein interaction networks.

Recent data have suggested that the mechanism of repression is predominantly via decrease in mRNA target stability [66]. Thus, miRNA-targeted genes regulated via mRNA stability are inversely correlated with the expression of their miRNA regulators. We previously identified 2 clusters of mRNA, respectively up- and down-regulated in patients suffering from AF *vs* control volunteers [39] (Fig 3B). In order to identify miRNA-mRNA functional pairs we crossed the transcriptional data [39] with the list of genes predicted to be targeted by the 42 dysregulated miRNAs (Fig 3). We found that 44.5% of the up-regulated mRNAs (Fig 6A) and 55% of the down-regulated mRNA (Fig 6B) were predicted to be conserved targets of the altered miRNAs (at least one binding sites in 3’ UTR), strongly suggesting that dysregulations of specific miRNAs can explain a great part of the transcriptional defects previously detected in LA from AF patients [39]. The highly targeted mRNA (*i*.*e*. the top 100 genes of each lists, S4 Table) encoded proteins involved in 7 functional networks (Fig 6C and 6D) (*i*.*e*. TGF-beta IGF-1 and MAPKinase signaling pathways, cellular adhesion/contact, proliferation and microtubule dynamic/ion fluxes, and mRNA splicing and silencing).

### Experimental validation of miRNA-mRNA interactions

As our study was based on *in silico* analyses, we aimed at confirming that part of the altered transcriptional signature was the consequences of dysregulated miRNAs in LA from patients suffering from AF. For this we focused on *Smyd2* (SET and MYND domain containing 2), which was among the top 10 genes with the highest fold changes, in LA from AF patients vs controls [39]. SMYD2 is a protein-lysine N-methyltransferase that methylates both histones and non-histone proteins and is dispensable for heart development in mice [67]. SMYD2 is a cardioprotective protein which, through the methylation of p53, can prevent cell death [68].

**Fig 7:**
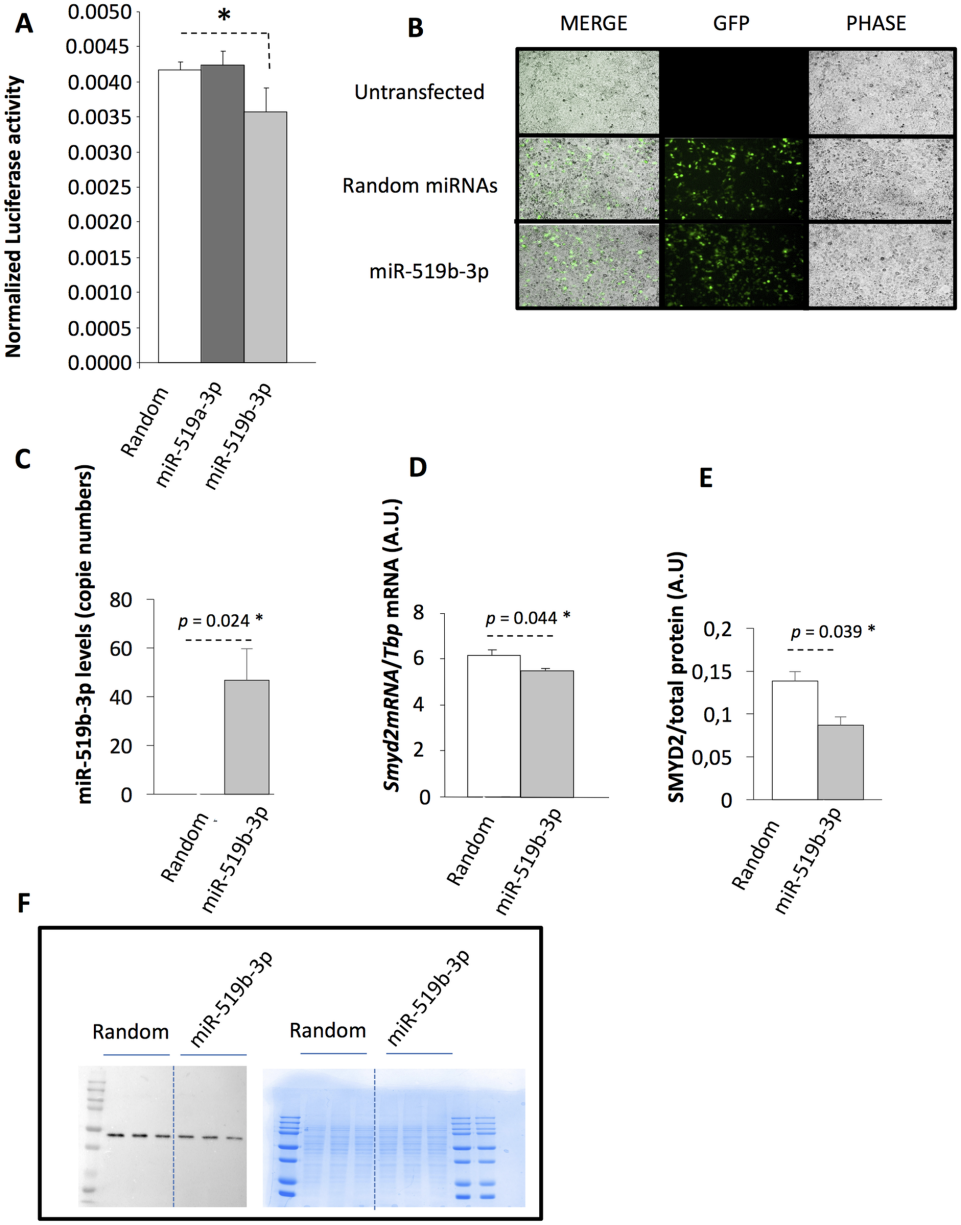
Validation of Smyd2 mRNA interaction with miR-519. A) HEK293T cells were transfected with reporter plasmid containing the 3’-UTR region of *Smyd2* associated with luciferase/renilla and with either a plasmid expressing random miRNAs, or pre-miR-519a-3p or pre-miR-519b-3p. B) Transfection efficiency with miR-519b-3p was validated by visualizing GFP in HEK293T and was found similar to the efficiency with random miRNAs. C) Validation of miR-519b-3p overexpression in HEK293T. D) Quantification of *Smyd2* mRNA level in HEK293T transfected a plasmid containing either pre-miR-519-3p or random miRNA sequences. E) Quantification of SMYD2 protein level in HEK293T transfected with either pre-miR-519-3p or random miRNA sequences. F) Western-blot of HEK293T protein extract, transfected either with pre-miR-519-3p or random miRNA sequences. Left, after blotting with anti-SMYD2 antibodies, right, acrylamide gel revealed with coomassie blue to validate that the quantity of protein loaded was the same for each sample.

The 3’-UTR regions of the 2 *Smyd2* human transcripts were predicted to be bind by miR-519a-3p and miR-519b-3p, 2 highly down-regulated miRNAs in LA (S3 Table). In order to validate these predictions, a reporter plasmid containing the 3’-UTR region of *Smyd2* associated with luciferase/renilla was co-transfected with either pre-miR-519a-3p or pre-miR-519b-3p. As shown on Fig 7A, only miR-519b-3p could bind the 3’-UTR region of *Smyd2* and reduce significantly the luciferase/renilla activity. In order to validate the down-regulation of *Smyd2* at the mRNA level, pre-miR-519b-3p was transfected in HEK293T. GFP detection in HEK293T was used to confirm the transfection efficiency (Fig 7B), and quantification of miR-519b-3p in HEK293T recipient cells confirmed its strong over-expression (Fig 7C). As shown on Fig 7D, miR-519b-3p overexpression lead a modest but significant down-regulation of *Smyd2* mRNA, compared with random miRNAs overexpression and a down-regulation of SMYD2 at the protein level (Fig 7E and 7F). These data confirmed that altered basal expression levels of miRNAs in LA from AF patients could participate in mRNA dysregulation [39]. In a previous study, we have demonstrated that epigenetic mechanisms (*i*.*e*.; promoter hypermethylation) contributed to the development of AF and were responsible for transcriptomic alterations in LA or pulmonary vein- LA junction from patients suffering from valvular heart disease [39]. The fact that miRNAs are associated with *Smyd2* altered expression in AF demonstrated the complex interplay between DNA methylation, histone modifications, and microRNAs in AF pathology.

## Discussion

AF is associated with the dilated atria of patients with VHD and contributes to worsened the pathology. Among the potential factors which may contribute to the development and perpetuation of AF, dysregulation of miRNAs had been suggested using biopsies from right atrial appendages [69, 70]. Surprisingly, although 90% of AF comes from LA, specially from the PVs, it was found that AF was associated with alteration of miRNA basal expressions in right atria (RA) but not in LA, by comparing patients suffering from VHD with or without AF [69]. The authors of this study postulated that this could be associated with tissue availability. In agreement, a more recent study gave opposite conclusions and showed that the development of AF was associated with changes in miRNA expressions both in RA and LA tissues [48]. In the present study, we have quantified the level of 662 miRNAs in patients undergoing surgery for mitral valve disease (MVD) with chronic AF (> 1 year) and compared with patients with MVD but without AF, focusing on the LA pathology only. Among the 299 miRNAs expressed in LA from patients with MVD and detected by qRT-PCR, 255 had been previously identified using microarrays, confirming their expressions in human LA. Among the 299, we found 42 miRNAs with altered basal expressions supporting the conclusions that AF is associated with altered miRNA levels in LA also [48]. They were predicted to regulate 35% of the coding genome, therefore confirming that AF is a miRNA-related disease. As the 299 miRNAs identified in this study matched only partly the 213 miRNAs identified previously in LA tissue (48), only five miRNAs were found commonly altered in both studies (*i*.*e* miR-378a-3p, miR-15b-5p, miR-21-5p, miR-125b-5p, and miR-451a). The 42 altered miRNAs were predicted to target cellular pathways known to be involved in AF (*i*.*e*. electrical remodeling, and signaling pathways characteristics of structural remodeling and hypertrophy development) further supporting their importance in the aetiology of the pathology. However, the raison for their altered expression in LA from AF patients is not clear and would deserve further studies. We can envisage 3 possible mechanisms (or a combination of the 3), such as promoter region hypermethylation, dysregulation of transcription factors involved either in their regulation or in the regulation of their host genes. But as the majority of miRNA promoter regions have not been identified yet it is difficult to conclude. Also, our analyses did no permit to determine whether expression of these miRNAs correlated with that of their corresponding pri-miRNAs.

Conversely to previous studies, which based their conclusions on the analysis of the functions predicted for individual miRNAs [36, 38], the originality of our study relies on the fact that we have considered the collective action of miRNAs in order to identify the most relevant genes and protein networks involved in AF. Indeed, it has been demonstrated that the decrease of target expression by individual miRNAs is usually quite modest [71, 72] and is confirmed here with the study of the interaction between *Smyd2* and miR-519b-3p. Moreover, miRNA-target predictions give false positives across all prediction methods. To try to overcome these biases, we have decided to consider only miRNA-binding site predictions conserved among species [43] and to focus on the 100 genes being the most targeted by either the up- or down-regulated miRNAs. Following this procedure, we were able to retrieve the most affected cellular pathways in which all partners of the networks are collectively under the control of the 42 dysregulated miRNAs in AF. They were involved in several important biological processes and functional pathways associated with AF, such as TGB-beta 1 and IGF-1 signaling, proliferation and cellular adhesion (MAPkinase and AKT pathways), and circadian clock/synchronisation of circadian rhythmicity. Indeed, the TGB-beta 1 pathway is regarded as one of the profibrotic mediator that cause selective atrial fibrosis and promote AF [73]. Low IGF-I serum levels are independently associated with AF in elderly population [74] and its antiarrhythmic properties have been shown on a rat model with ventricular arrhythmia [75]. IGF-I protects against atherosclerosis, counteracts endothelial dysfunction, and prevents smooth muscle cell apoptosis [76]. Furthermore, IGF-I reduces proinflammatory cytokine production [76] and oxidative stress [77]. This may have relevance because inflammation, oxidative stress, and endothelial dysfunction are involved in AF aetiology [78–80]. In addition, bioinformatics predictions identified protein-protein interaction networks involved in circadian rhythm and in the regulation of circadian rhythmicity, as highly controlled by miRNAs in LA. It has been shown that alteration of circadian rhythm can induce the development of cardiac diseases (*e*.*g*.; AF exhibits a circadian rhythm with a double peak [81]) and that its resynchronisation could become a promising therapeutic target to prevent disease progression [82]. With our approach, we were able to identify a network of proteins involved in synchronisation of circadian rhythmicity predicted to be collectively under the control of the altered miRNAs in LA from AF patients. CREB1, which was the most connected protein of the protein network, had been previously predicted as a target gene of miRNAs altered in AF patients [7, 38, 83]. Interestingly it has been found that CREB proteins expressions, which are undetectable in myocytes from young rat increased dramatically with age [84], which is the biggest risk factor for AF and for perturbation of circadian rhythm. An other study demonstrated a cytoprotective role of CREB when cells are subjected to oxidative stress [85], a condition associated to AF [79]. Therefore increasing CREB1 and its partner through the down-regulation of specific miRNAs, may represent a kind of protection/adapation for cell surviving during the development of AF. Finally, our *in silico* data strongly suggest that miRNAs functioning (AGO proteins, TNRC6B and TNRC6A) is perturbated in AF, as targeted by multiple dysregulated miRNAs. It has been shown that the expression level of AGO proteins in heart appears to be lower than those in other tissues [86] suggesting a tight control of their expressions by miRNAs. As consequences, decreased expression of miRNAs in AF might increase their levels and dramatically perturbate miRNA functioning.

Finally, we tried to determine whether altered miRNAs levels might participate and explain the altered transcriptional signature in LA from AF patients [39]. Indeed, recent data have suggested that the mechanism of repression is also via decrease in mRNA target stability [66, 87]. Our analyses predicted that 44.5% of the up-regulated mRNAs and 55% of the down-regulated mRNA were conserved targets and were inversely expressed to the altered miRNAs. There were no correlations between the number of binding sites in the 3’-UTR region and the mRNA fold changes. It has to be noticed that our analyses, based on TARGETSCAN predictions, did not take into account miRNA binding sites in the coding region of mRNAs. Highly targeted genes involved in TGF-beta and IGF-I pathways and miRNA functioning were predicted to be regulated at the transcriptional level, suggesting that these 3 pathways would be regulated predominantly via decreasing mRNA levels during AF.

Taken together, this study provides evidence that miRNAs are part of the molecular alterations in LA occurring in patients with atrial remodeling and hypertrophy associated with AF. If there is no doubt that miRNAs can be used as diagnostic biomarker or to monitor the effect of therapy, our results suggest that selective miRNA target therapy, as either up-regulation by adenovirus transfection or a down-regulation by antagomiR, is likely unfeasible to treat AF patients. Because miRNAs act collectively on gene expression and regulate many overlapping pathways at the same time the selection of one specific miRNAs might be not enough to restore LA homeostasis. Moreover, it is not clear whether alterations of miRNA levels in AF pathology are causal (early event in the pathology) or represent a sort of adaptation to fight against cardiac electrical remodeling and fibrosis development.

## Supporting information

S1 Table List of the 299 miRNAs expressed in human left atrium and pulmonary vein- LA junction. (XLSX)

S2 Table Number of miRNA binding sites in 3'-UTR of the genes predicted to be targeted by the 299 commonly expressed miRNA in human left atrium (n = 8). Prediction are from TARGETSCAN (http://www.targetscan.org/vert_70).
(XLSX)

S3 Table List of miRNA differentially expressed in left atria of patients suffering from AF. miRNA I.D are from http://www.mirbase.org/; miRNA host gene determinations are from http://mirstart.mbc.nctu.edu.tw/browse.php. Host gene fold changes are from Doñate Puertas R et al. Transl Res. 2017 Jun;184:57–67.
(XLSX)

S4 Table Targets of dyregulated miRNAs in left atria from patient suffering from atrial fibrillation. Predictions are from TARGETSCAN 6.2 (http://www.targetscan.org/vert_61/).
(XLSX)

## Abbreviations

AF: atrial fibrillation
BMI: body mass index
Ct: threshold cycle
HEK293T: human embryonic kidney 293T cell line
IGF-1: Insulin-like growth factor 1
LA: left atrium
miRNA: microRNA
MVD: mitral valve disease
PCR: polymerase chain reaction
PV: pulmonary vein
RA: right atrium
TBP: TATA box binding protein
TGF: Transforming growth factor
VHD: valvular heart disease
